# Isolation, bioassay and 3D-QSAR analysis of 8-isopentenyl flavonoids from *Epimedium sagittatum* maxim. as PDE5A inhibitors

**DOI:** 10.1186/s13020-022-00705-5

**Published:** 2022-12-31

**Authors:** Juntao Li, Yue Wu, Xinxin Yu, Xinyu Zheng, Jiechen Xian, Senjie Li, Wanyin Shi, Yun Tang, Zhe-Sheng Chen, Guixia Liu, Shen Yao, Jian Xu, Xiangwei Zheng

**Affiliations:** 1 Engineering Research Center of Modern Preparation Technology of Traditional Chinese Medicine, Ministry of Education, Innovation Research Institute of Traditional Chinese Medicine, Shanghai University of Traditional Chinese Medicine, No. 1200 CaiLun Road, Pudong District, Room 10112, Shanghai, People’s Republic of China; 2 Hubei Provincial Key Laboratory for Quality and Safety of Traditional Chinese Medicine Health Food, Jing Brand Research Institute, No.169 Daye Ave, Daye, Jing Brand Co. Ltd, Huangshi, Hubei People’s Republic of China; 3 Shanghai Key Laboratory of New Drug Design, School of Pharmacy, East China University of Science and Technology, 130 Meilong Road, Box 318, Shanghai, 200237 China; 4 Sanlin Community Health Service Center of Shanghai Pudong New District, No. 375 Sanlin Road, Shanghai, 200124 China; 5 Department of Pharmaceutical Sciences, College of Pharmacy and Health Sciences, St. John’s University, New York, 11439 USA

**Keywords:** 8-isopentenyl flavonoid, The processed folium of *Epimedium sagittatum* Maxim., Phosphodiesterase-5 inhibitor, 3D-QSAR, cGMP-PKG-Ca^2+^ signaling pathway

## Abstract

**Background:**

As known, inhibition of phosphodiesterase 5 (PDE5) has the therapeutic effect on male erectile dysfunction (ED), and the processed folium of *Epimedium sagittatum* Maxim. (PFES) characterized by 8-isopentenyl flavonoids is a famous herb for treating ED. However, the main flavonoids inhibitory activities, structure–activity relationship (SAR) and signaling pathway have been not systematically studied so that its pharmacodynamic mechanism is unclear.

**Methods:**

We aimed to initially reveal the PFES efficacy mechanism for treating ED. For the first time, 6 main 8-isopentenyl flavonoids (1–6) from PFES were isolated and identified. Then based on HPLC detection, we proposed a novel method to screen inhibitors among them. We further analyze the three-dimensional quantitative structure–activity relationship (3D-QSAR) for those inhibitors.

**Results:**

The results were verified by cellular effects of the screened flavonoids. Among 6 compounds, Icariin: (1), 2-Oʹʹrhamnosylicaridide II (2) and Baohuoside I (3) were identified with significant activities (IC_50_ = 8.275, 3.233, 5.473 μM). Then 3D-QSAR studies showed that the replacement of C8 with bulky steric groups as isopentenyl, C3 with positive charge groups and C4' with a hydrogen bond acceptor substituent could increase inhibitory effects. In contrast, the substitution of C7 with bulky steric groups or hydrophilic groups tended to decrease the efficacies. And compounds 1, 2, 3 could increase cGMP level and decrease cytoplasmic Ca^2+^ of rat corpus cavernosum smooth muscle cells (CCSMCs)by activating PKG.

**Conclusion:**

8-isopentenyl flavonoids could be the main pharmacodynamic substances of PFES in the treatment for ED, and some had significant PDE5A1 inhibitory activities so as to activate cGMP/PKG/Ca^2+^ signaling pathway in CCSMCs, that was related to the substituents at the key sites such as C8, C3, C4ʹ and C7 in the characteristic compounds.

**Supplementary Information:**

The online version contains supplementary material available at 10.1186/s13020-022-00705-5.

## Introduction

Erectile dysfunction (ED), defined as the inability of the penis to achieve or maintain sufficient erections, is one of the most common male sexual disorders [1]. Phosphodiesterase 5 (PDE5) is highly expressed in corpus cavernosum and has become a noteworthy target for drug screening in ED [2, 3]. The PDE5 inhibitors relax the penile blood vessel by blocking the hydrolyzation of the second messenger cGMP. Clinically, admission of PDE5 inhibitors is the main treatment for ED. However, in specific cases, drugs containing PDE5 inhibitors were not applicable to patients, otherwise disease progression might happen, such as sildenafil could not be prescribed to patients with severe cardiovascular diseases [4]. In addition, the established side effects of PDE5 inhibitors in the market include headache, dyspepsia, vomiting, diarrhea, and vision disorders [5]. Compared to synthetic compounds, naturally derived compounds, becoming an important resource for new drugs, might exhibit higher safety value, unique pharmacological properties, and less side effects.

The processed folium of *Epimedium sagittatum* (Sieb. et Zucc) Maxim. (PFES), Yinyanghuo in Chinese, which is soaked in mutton oil and fried for use, is one of the most commonly used traditional Chinese medicines to reinforce the kidney Yang. In modern pharmacological research, the PFES showed ability to strengthen sexual function and treat ED [6–8]. The underlying mechanism was proposed that it might relax the corpus cavernosum smooth muscle through activation of multitargets on NO/PDE5/cGMP signaling pathway [9]. The characteristic chemical components of PFES are8-isopentenyl flavonoids, of which 6 compounds are high in content, including icariin, baohuoside I, epimedin A, B, C and 2-O"-rhamnosylicaridide II [10]. However, until now, it was only reported that icariin exhibited an IC_50_ value of 5.9 μM against PDE5A1 in vitro [11], the 6 components have not been separated from PFES and the PDE5 inhibitory potencies have not been scrutinized. Moreover, there is few SAR study on flavonoids containing bulky substituents like isopentenyl at C8position as PDE5 inhibitors.

Compared with the detection for cGMP level using isotope labeling or fluorescent labeling, HPLC is simpler and easier to operate. Because of the fact that HPLC could detect the reduction of cGMP caused by PED5 hydrolysis, the combination of enzyme reaction activity and HPLC could be applied to analyze the activity of 8-isopentenyl flavonoids from PFES. Although recent studies presented various computational methods for drug design and discovery, 3D-QSAR analysis is still one of the most prominent and well-developed approaches, especially in predicting the activities of novel compounds and the optimization of lead compounds [12]. Moreover, based on PDE5 inhibitory mechanism, the effect and mechanism study on ED could be evaluated using cavernous smooth muscle cells (CSMCs) cellular assay in vitro. In this study, for the first time, 6 main 8-isopentenyl flavonoids, Icariin: (1), 2-O"-rhamnosylicaridide II (2), Baohuoside I (3), Epimedin A, B, C (4–6) were separated from the PFES and identified by ^1^H NMR, ^13^C NMR and MS, and these compounds were detected PDE5A1 inhibitory activity with the novel HPLC assay. We also adapted a series of PDE5 inhibitors from different reports [11, 13] to firstly establish CoMFA and CoMSIA models, aiming at finding the relationship between the biological activity and the key structural factors of 8-isopentenyl flavonoids from the models. Furthermore, The SAR results were verified by the cellular effects of the cGMP level and cytoplasmic Ca^2+^ in CSMCs, and the mechanism on PKG/Ca^2+^ signaling pathway was also studied.

## Materials and methods

### Plant material

The leaves of refined mutton fat-PFES were purchased and identified by Dr. *Jian Xu* in Jing Brand Co., Ltd., Hubei Province, China, in May 2019. A specimen voucher (20190501) had been stored in the Hubei Provincial Key Laboratory for Quality and Safety of Traditional Chinese.

### Chemicals, reagents, and apparatus

PDE5A (ab80330), cGMP, Rp-8-Br-cGMPS, MgCl_2_, Tris–HCl and DMSO were all purchased from Sigma-Aldrich (Merck KGaA, Darmstadt, Germany).

The HPLC system consisted of the Agilent 1200 instrument equipped with a quaternary pump (G1311A, CA, USA), an autosampler (G1329A), a Unitary C18 5 μm reversed phase analytical column (250 × 4.6 mm, Wenling, China), a thermostatted column compartment (G1316A), a diode ultraviolet detector (G1314B) operated at 254 nm, and the analytical software for data acquisition (ChemStation B.04.01, CA, USA). NMR spectra were recorded on Bruker 600 NMR spectrometer, and TMS was used as internal standard. ESI–MS data were obtained on Agilent Series 1100 SL mass spectrometer. NS4000 System with C18 20 × 250 mm 10 μm preparative columns. Column chromatography (CC) was performed with silica gel (200−300 mesh, Qingdao Marine Chemical Inc., Qingdao, China). Sephadex LH-20 (Pharmacia Biotech, USA) was used for column chromatography. All solvents used for HPLC were HPLC grade solvents and all solvents used for extraction and chromatography separation were analytical grade solvents.

### Extraction and isolation

The powdered leaves of refined mutton fat-PFES (25 kg) were extracted with 50% ethanol reflux for 2 h. The extraction was repeated twice. The filtrate was mixed and vaporized under low pressure to obtain ethanol extract (5 kg). The extract was chromatographied over Macroporous adsorption resin LSA-12S, eluted with 20%, 40%, 60%, 80% and 95% ethanol in successive. The 60% ethanol eluate (305 g) was exacted with petroleum, CH_2_Cl_2_, EtOAc, successively. CH_2_Cl_2_ layer (3 g) was subjected to 6010-C18 eluted with 55% MeOH-H_2_O, and then separated by preparative HPLC (C18, 10 μm, 20 × 250 nm, flow rate 19 ml/min) with (ACN/H_2_O gradient from 10:90 to 90:10) to give compound **4** (3.28 mg, t_R_ 12.6 min). Column chromatography (CC) was performed on silica gel (200–300 mesh, 3 kg,180 × 200 mm) with EtOAc layer (160 g) eluted with methylene chloride methanol (25:1,15:1,5:1,0:1, 25 L each) to obtain fractions. Fraction 1 (CH_2_Cl_2_/MeOH 25:1) was separated by preparative HPLC (C18, 10 μm, 20 × 250 nm, flowrate 19 ml/min) with (28% acetonitrile) to yield compound 5 (4.3 mg, t_R_ 10.6 min). Fraction 2 (CH_2_Cl_2_/MeOH 15:1) was subjected to 6010-C18 eluted with 80% MeOH-H_2_O, and then separated by preparative HPLC (C18, 10 μm, 20 × 250 nm, flow rate 19 ml/min) with (45% acetonitrile) to give compound 3 (3 g, t_R_ 12.2 min). Fraction 3 (CH_2_Cl_2_/MeOH 5:1) was subjected to 6010-C18 eluted with 80% MeOH-H_2_O, and then separated by preparative HPLC (C18, 10 μm, 20 × 250 nm, flow rate 19 ml/min) with (ACN/H_2_O gradient from 30:70 to 90:10) to yield compounds 1 (130 mg, t_R_ 6.7 min) and 2 (200 mg, t_R_ 13.2 min). Fraction 4 (CH_2_Cl_2_/MeOH 10:1) was subjected to 6010-C18 eluted with 80% MeOH-H_2_O, and then separated by preparative HPLC (C18, 10 μm, 20 × 250 nm, flow rate 19 ml/min) with (28% acetonitrile) to give compound 6 (5.95 mg, t_R_ 11.8 min).

### Chromatographic conditions

50 μL analytes were injected and separated on the C18 5 μm column with a flow rate of 1 mL/min at 30 °C column temperature. The mobile phase consisted of methanol and 0.05 mol/L potassium dihydrogen phosphate (10:90, V/V). The absorption was detected at 254 nm wavelength.

### PDE5A1 inhibition assays

All components for assay were diluted in assay buffer which contained 100 mM MgCl_2_ and 50 mM Tris–HCl (pH 8.0). The compounds were dissolved in DMSO and then further diluted to the desired concentration with assay buffer. 30 μL PDE5A1 solution and 30 μL compound solution were mixed at room temperature and incubated for 5 min, followed by addition of with 30 μL cGMP solution, and the reaction was carried out at 35 °C for 90 min. After the assay, each vial was incubated in 100 °C boiling water for 5 min to inactivate the PDE5A1 activity, and then was cooled to be detected with HPLC. No PDE5A1 was used as the positive control, and DMSO was used as the negative control. Percentage inhibition was calculated using the peak area of HPLC with the following formula.

For dose–response experiments, the PDE5A1 inhibitors were diluted to 7 concentrations series with fourfold gradient. Two individual operations were performed on different dates as the repetition.

### Precision, stability and reproducibility assessment

The precision of HPLC was determined by spiking blank samples with 7.5 mg/mL cGMP (n = 6). To demonstrate the analytes stability, the analytes were placed at room temperature for 0 ~ 24 h after PDE5A1 inhibition by 10 nM sildenafil, then were injected into the analytical column for analysis. The PDE5A1 inhibition analysis by 10 nM sildenafil was repeated six times to verify the reproducibility of the assay analytical performance. The relative standard deviation (RSD) was calculated to access the analytical performance.

### SAR dataset

A dataset comprising 17 PDE5A1 inhibitors was used as model dataset. The potencies of compounds 1–6 and 16 were obtained by our in vitro activity assay combined with HPLC. The remains were adapted from two literature [11, 13]. Although these compounds were taken from different sources, the compound 16 [11, 13] showed similar values of inhibitory concentration (IC_50_) with ours, 0.075, 0.010 and 0.014 (our result) μM, respectively.

The structures of the molecules and their biological data were given in Table 1. IC_50_ of the molecules were converted into corresponding pIC_50_ (-logIC_50_). The total set of inhibitors was randomly divided into a training set (14 compounds) for generating 3D-QSAR model and a test set (3 compounds) for validating the quality of the model. The biological activity of test set molecules contains a range of low, moderate and high activity.

**Table 1:** Lists of structures including studied molecules and corresponding experimental activity
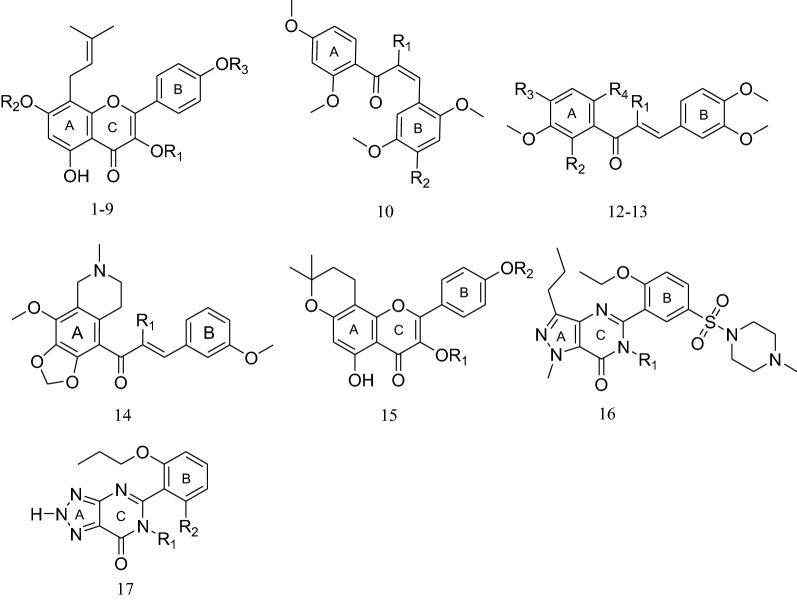

Compd	R_1_	R_2_	R_3_	R_4_	pIC_50_
1	rha	glu	CH_3_		5.082
2	rha- (1–2) rha	H	CH_3_		5.490
3	rha	H	CH_3_		5.262
4^t^	rha- (1–2) glu	glu	CH_3_		3.389
5	rha- (1–2) xyl	glu	CH_3_		3.447
6	rha- (1–2) rha	glu	CH_3_		3.166
7	H	H	CH_3_		5.658
8	rha	CH_2_CH_2_OH	CH_3_		6.444
9	CH_2_CH_2_OH	CH_2_CH_2_OH	CH_3_		7.131
10^t^	H	OCH_3_			5.745
11	H	H			5.222
12^t^	H	OCH_3_	OCH_3_	OCH_2_Phe	5.229
13	H	H	H	OCH_3_	5.155
14	H				5.208
15	H	CH_3_			4.342
16	H				7.847
17	H	H			6.222

*t* test set compounds

### Molecular modeling and 3D-QSAR analyses

All molecular modeling and calculations were performed by the Sybyl program package [14]. The structures of all compounds were constructed using the Sketch Molecule module. Structural energy minimization was carried out using the standard Tripos molecular mechanics force field and Gasteiger-Huckle charge, with the convergence criterion set at 0.005 kcal/(Å·mol) and the max iterations for the minimization set to 10,000.

#### Molecular alignment

**Fig. 1:**
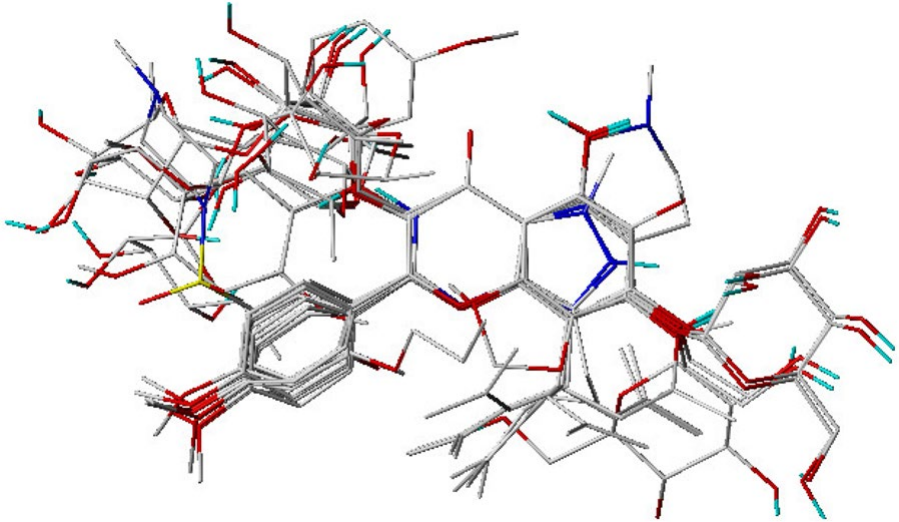
Alignment of the compounds used as the training set

Because the quality and the predictive ability of the 3D-QSAR model rely directly on the alignment rules, we aligned the active conformations of the compounds derived by energy minimization previously. Compound 16 was chosen as the template molecule to fit the remaining training set and test set compounds by using the Database Align function in Sybyl [14]. Alignment of compounds in the training set was shown in Fig. 1.

#### CoMFA

The 3D cubic lattice region was created automatically and the grid spacing was set at 1.7 Å in the x, y, and z directions. A sp^3^ carbon atom with + 1 net charge was taken as the probe atom and steric and electrostatic interactions were calculated by using the Tripos force field. A cutoff of 30 kcal/mol was adopted in calculating energies. With standard options for scaling of variables, the regression analysis was carried out using partial least squares (PLS) method [15]. The cross-validation with Leave-One-Out (LOO) [16] option was carried out to obtain the optimum number of components (ONC).The final model (non-cross-validated conventional analysis) was developed with the optimum number of components. Region focusing is an advanced strategy that improves prediction ability of the initial built CoMFA model. CoMFA region focusing method refines a model by increasing the weights of the lattice points. StDev × coefficient values were used as weights.

#### CoMSIA

Although CoMSIA is similar to CoMFA, CoMSIA not only computes the steric and electrostatic fields, but also calculates additional hydrophobic, hydrogen-bond donor, and hydrogen-bond acceptor fields. CoMSIA descriptors were calculated with the same lattice box, grid spacing and probe atom as that used for the CoMFA calculations. In CoMSIA analysis, the five different descriptor fields are not totally independent from each other. Given that the size of dataset, we decided to use three of five fields as the descriptors.

### Isolation and culture of rat corpus cavernosum smooth muscle cells (CCSMCs)

Male Sprague–Dawley (SD) rats (180–220 g) were purchased from Shanghai SLAC Laboratory Animal Co., Ltd. This experiment protocol was approved by the standardizing laboratory animal ethical review of Shanghai University of Traditional Chinese Medicine. After removal of the corpus spongiosum, subcutaneous tissue and deep fascia of penises of SD rats, the corpus cavernosum tissue was cut into small pieces of less than 2mm^3^ and digested with 0.5% collagenase I and 20% fetal bovine serum (PFES) in DMEM for 4 h. The cell suspensions were collected by mesh filtration and centrifugation, and cultured in DMEM containing 20% PFES and 1 × antibiotics at 37 °C in a humidified atmosphere with 5% CO_2_. After 24 h culture, spindle CCSMCs attached to the culture dish. The medium was replaced with DMEM containing 10% PFES when CCSMCs reached 70% confluence.

### CCK-8 assay

A CCK-8 assay with 5,000 cells per well and five replicates in 96-plate was used to determine the toxic effects of 1 ~ 3 of 2.5 ~ 10 μM after 48 h. The inhibition rate was determined.

### cGMP assay

CCSMCs were seeded in 6-well plate at a density of 100,000/well for 24 h, and then were treated with 10 μM 1 ~ 3 for 48 h. The cells were lysed by 0.1 M hydrochloric acid with 200 μl per well for 20 min incubation, then the supernatant was collected and determined the cGMP content by ELISA.

### Fluorescent staining of calcium

CCSMCs were cultured in 6-well plate at a density of 100,000/well for 48 h. Then cells were stained by Fluo-4AM for 30 min prior to 10 mΜ 1 ~ 3 exposure for 30 min in the presence or absence of 10 μM Rp-8-Br-cGMPS (the protein kinase G inhibitor). After PBS rinse, the cytosolic calcium was observed by fluorescence microscope.

## Results and discussion

### Structure elucidation

Compound 1. Yellow amorphous powder. ESI–MS: m/z = 677.52 [M + H]^+^. The ^1^H and ^13^C NMR data were generalized in Tables 2 and 3, and the data were consistent to the reported literature [17]. It was elucidated as icariin.

**Table 2:** ^1^H NMR Data of Compounds 1–6 (600 Hz, in CD_3_OD)

Position	1	2	3	4	5	6
6	6.64 (1H, *s*)	6.26 (1H, *s*)	6.24 (1H, *s*)	6.63 (1H, *s*)	6.64 (1H, *s*)	6.65 (1H, *s*)
12	5.18 (1H, *t*, *J* = 7.1)	5.19 (1H, *t*, *J* = 6.9)	5.16 (1H, *t*, *J* = 7.2)	5.17 (1H, *t*, *J* = 7.0)	5.17 (1H, *t*, *J* = 7.0)	5.19 (1H, *t*, *J* = 6.6)
14	1.63 (3H, *s*)	1.66 (3H, *s*)	1.64 (3H, *s*)	1.69 (3H, *s*)	1.70 (3H, *s*)	1.72 (3H, *s*)
15	1.71 (3H, *s*)	1.71 (3H, *s*)	1.70 (3H, *s*)	1.60 (3H, *s*)	1.62 (3H, *s*)	1.64 (3H, *s*)
2ʹ 6ʹ	7.87 (1H, *d*, *J* = 8.7)	7.87 (1H, *d*, *J* = 8.9)	7.84 (1H, *d*, *J* = 7.8)	7.89 (1H, *d*, *J* = 8.5)	7.90 (1H, *d*, *J* = 8.9)	7.86 (1H, *d*, *J* = 8.4)
3ʹ 5ʹ	7.09 (1H, *d*, *J* = 8.6)	7.09 (1H, *d*, *J* = 8.9)	7.07 (1H, *d*, *J* = 7.9)	7.13 (1H, *d*, *J* = 8.5)	7.15 (1H, *d*, *J* = 8.9)	7.08 (1H, *d*, *J* = 8.4)
4ʹ–OCH_3_	3.89 (3H, *s*)	3.89 (3H, *s*)	3.87 (3H, *s*)	3.85 (3H, *s*)	3.86 (3H, *s*)	3.89 (3H, *s*)
3-O-rha						
1	5.40 (1H, *d*, *J* = 1.7)	5.40 (1H, *d*, *J* = 1.7)	5.38 (1H, *d*, *J* = 1.7)	5.55 (1H, *d*, *J* = 1.7)	5.36 (1H, *d*, *J* = 1.7)	5.53 (1H, *d*, *J* = 1.7)
6	0.90 (3H, *d*, *J* = 6.0)	0.90 (3H, *d*, *J* = 6.0)	0.90 (3H, *d*, *J* = 6.0)	0.87 (3H, *d*, *J* = 6.0)	0.89 (3H, *d*, *J* = 6.4)	1.21 (3H, *d*, *J* = 6.1)
7-O-	glu			glu	glu	glu
1	5.06 (1H, *d*, *J* = 7.4)			5.06 (1H, *d*, *J* = 6.8)	5.08 (1H, *d*, *J* = 7.4)	5.07 (1H, *d*, *J* = 7.4)
3-O-rha-2-		rha		glu	xyl	rha
1		4.88 (1H, *d*, *J* = 1.2)		4.45 (1H, *d*, *J* = 7.8)	4.44 (1H, *d*, *J* = 7.6)	5.07 (1H, *d*, *J* = 7.4)
6						0.91 (3H, *d*, *J* = 4.7)

**Table 3:** ^13^C NMR Data of Compounds 1–6 (150 Hz, in CD_3_OD)

Position	1	2	3	4	5	6
2	159.3	158.7	158.7	157.3	157.5	159.2
3	136.5	136.3	136.2	134.7	135.0	136.5
4	180.1	179.8	179.8	178.4	178.6	180.0
5	162.1	163.3	163.3	160.5	160.8	162.1
6	99.34	99.4	99.42	98.1	98.4	99.37
7	163.5	163.4	163.4	161.4	161.8	163.5
8	110.5	107.9	107.9	108.3	108.6	107.5
9	155.0	155.8	155.8	153.0	153.3	155.0
10	107.5	105.9	106.0	105.6	106.7	103.7
11	22.73	22.4	22.4	21.4	21.7	22.7
12	123.5	123.7	123.7	122.1	122.4	123.6
13	132.6	132.5	132.4	131.1	130.8	132.6
14	25.87	25.9	25.9	25.5	25.8	25.9
15	17.66	17.8	17.6	17.5	17.7	17.8
1ʹ	123.9	124.1	124.1	122.3	122.3	123.8
2ʹ 6ʹ	131.9	131.8	131.8	130.6	131.5	131.9
3ʹ 5ʹ	115.2	115.2	115.1	114.1	114.5	115.2
4ʹ	161.1	160.8	160.8	159.1	159.4	161.0
4ʹ–OCH_3_	56.0	56.0	56.0	55.5	55.8	56.0
3-O-rha						
1	103.5	102.3	103.5	101.6	101.5	101.9
2	72.1	78.9	73.2	81.74	80.9	78.2
3	72.1	72.25	72.0	70.6	69.8	72.1
4	71.2	74.0	72.1	72.1	71.9	73.9
5	71.9	71.9	71.9	70.9	69.9	71.1
6	18.3	18.1	18.1	17.8	17.8	17.8
7-O- glu						
1	101.9			101.1	101.1	102.3
2	74.9			73.8	73.9	74.9
3	78.3			76.7	77.1	78.3
4	73.9			70.1	70.2	70.3
5	78.3			77.7	77.5	78.9
6	62.4			61.1	61.2	62.4
3-O-rha-2-		rha		glu	xyl	rha
1		103.7		106.1	106.0	103.7
2		71.9		74.3	74.2	71.9
3		72.0		77.7	76.7	72.2
4		73.5		69.8	70.9	73.5
5		70.3		77.1	66.3	71.9
6		17.9		60.9		17.8

Compound 2. Yellow amorphous powder. ESI–MS: m/z = 699.48 [M + K]^+^. The ^1^H and ^13^C NMR data were generalized in Tables 2 and 3, and the data were consistent to the reported literature [18]. It was elucidated as2-O"-rhamnosylicaridide II.

Compound 3. Yellow amorphous powder. ESI–MS: m/z = 515.30 [M + H]^+^. The ^1^H and ^13^C NMR data were generalized in Tables 2 and 3, and the data were consistent to the reported literature [19]. It was elucidated as baohuoside I.

Compound 4. Yellow amorphous powder. ESI–MS: m/z = 861.21 [M + Na]^+^. The ^1^H and ^13^C NMR data were generalized in Tables 2 and 3, and the data were consistent to the reported literature [20]. It was elucidated as epimedin A.

Compound 5. Yellow amorphous powder. ESI–MS: m/z = 809.11 [M + H]^+^, The ^1^H and ^13^C NMR data were generalized in Tables 2 and 3, and the data were consistent to the reported literature [20]. It was elucidated as epimedin B.

Compound 6. Yellow amorphous powder. ESI–MS: m/z = 823.03 [M + H]^+^, The ^1^H and ^13^C NMR data were generalized in Tables 2 and 3, and the data were consistent to the reported literature [20]. It was elucidated as epimedin C.

### Development of a screening assay for identifying PDE5A1 inhibitors

The development of PDE5 inhibitor, an important drug target for ED, has attracted much attention in the field. The common assays for screening PDE5 inhibitors use labeled cGMP as substrate, among which, ^3^[H] is the main isobaric tags [21–23]. Other detection methods using fluorescence detection, such as fluorescence resonance energy transfer (FRET) [24] and fluorescence polarization (FP) [25], are also used occasionally. Although well developed, handling radioactive isotype in lab environment is rigorous while commercial fluorescence kits are expensive. Owing to these concerns, it is of practical significance to develop a novel detection method which could be popularized, economic, and easy to operate. To answer the urgent need, HPLC, as a common, simple, and effective detection technique, could be used for analyte separation and content analysis. This developed HPLC assay would be conducive for most of ordinary laboratories for PDE5 inhibitors screening, and even further be applied to studies focusing on PDE5 for other disease treatment.

PDE5A1, PDE5A2 and PDE5A3 are three isoforms of PDE5 [26, 27], which have similar function and cGMP catalytic activity, and are inhibited with similar activity by sildenafil, but differ at their N-terminal regions [28]. Thus, in this present study, PDE5A1 was used as the representative PDE5 isoform in inhibitors screening from the PFES.

**Fig. 2:**
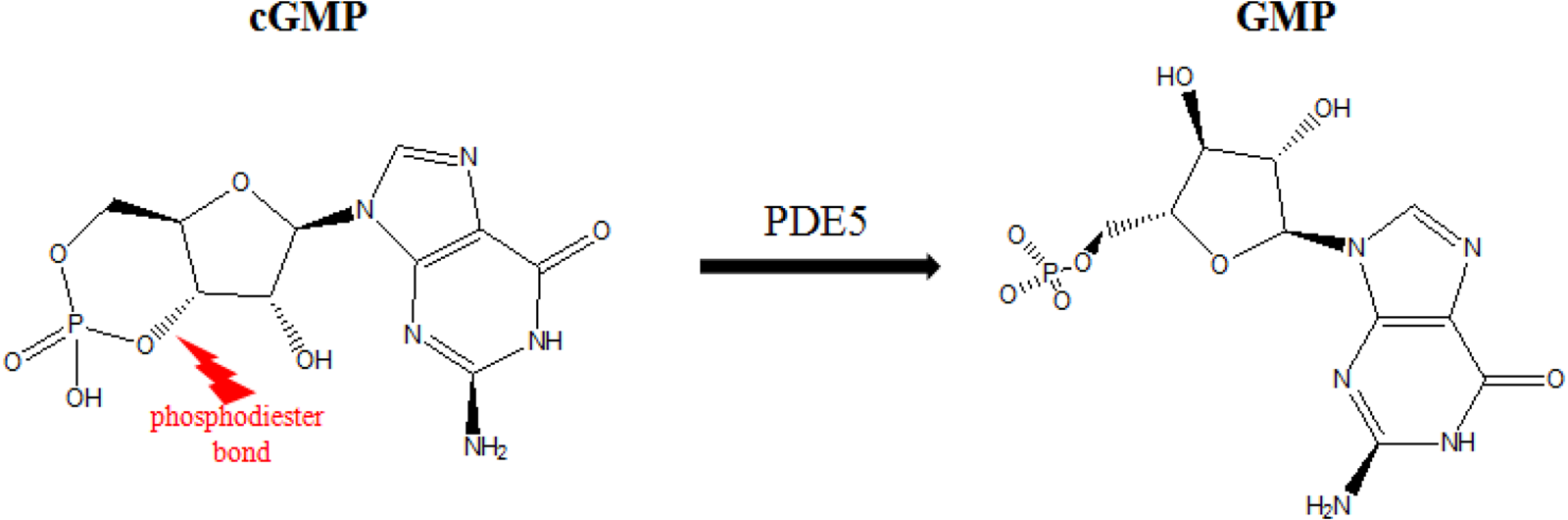
Hydrolysis of cGMP by PDE5

**Fig. 3:**
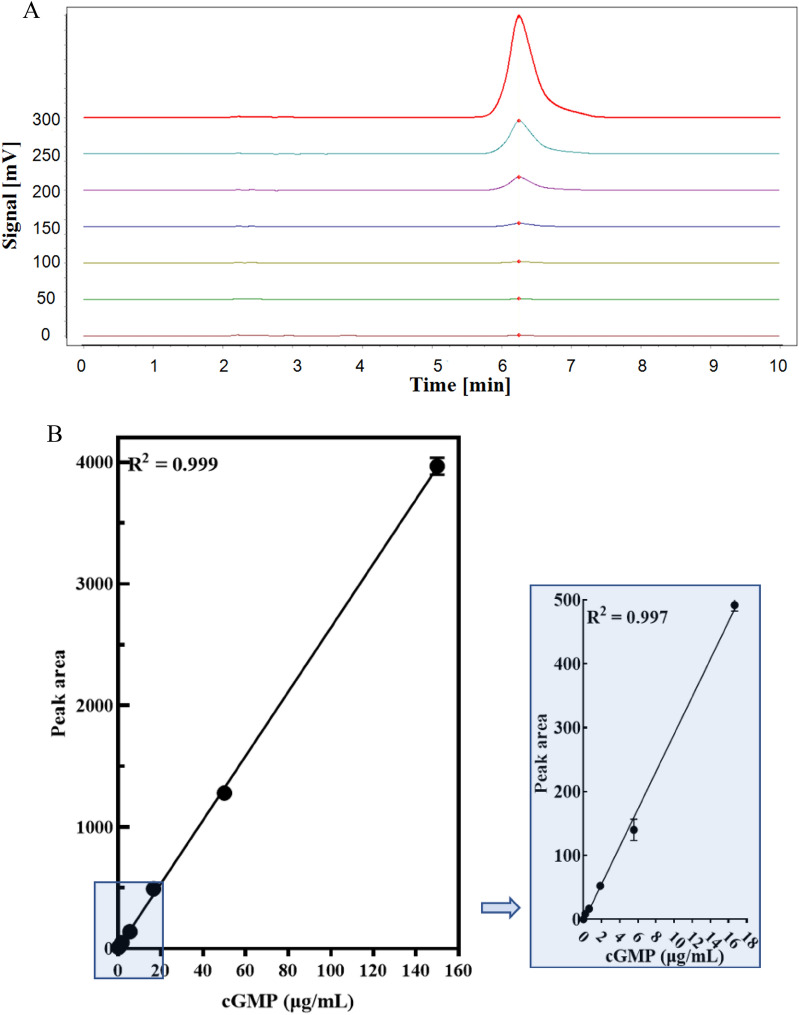

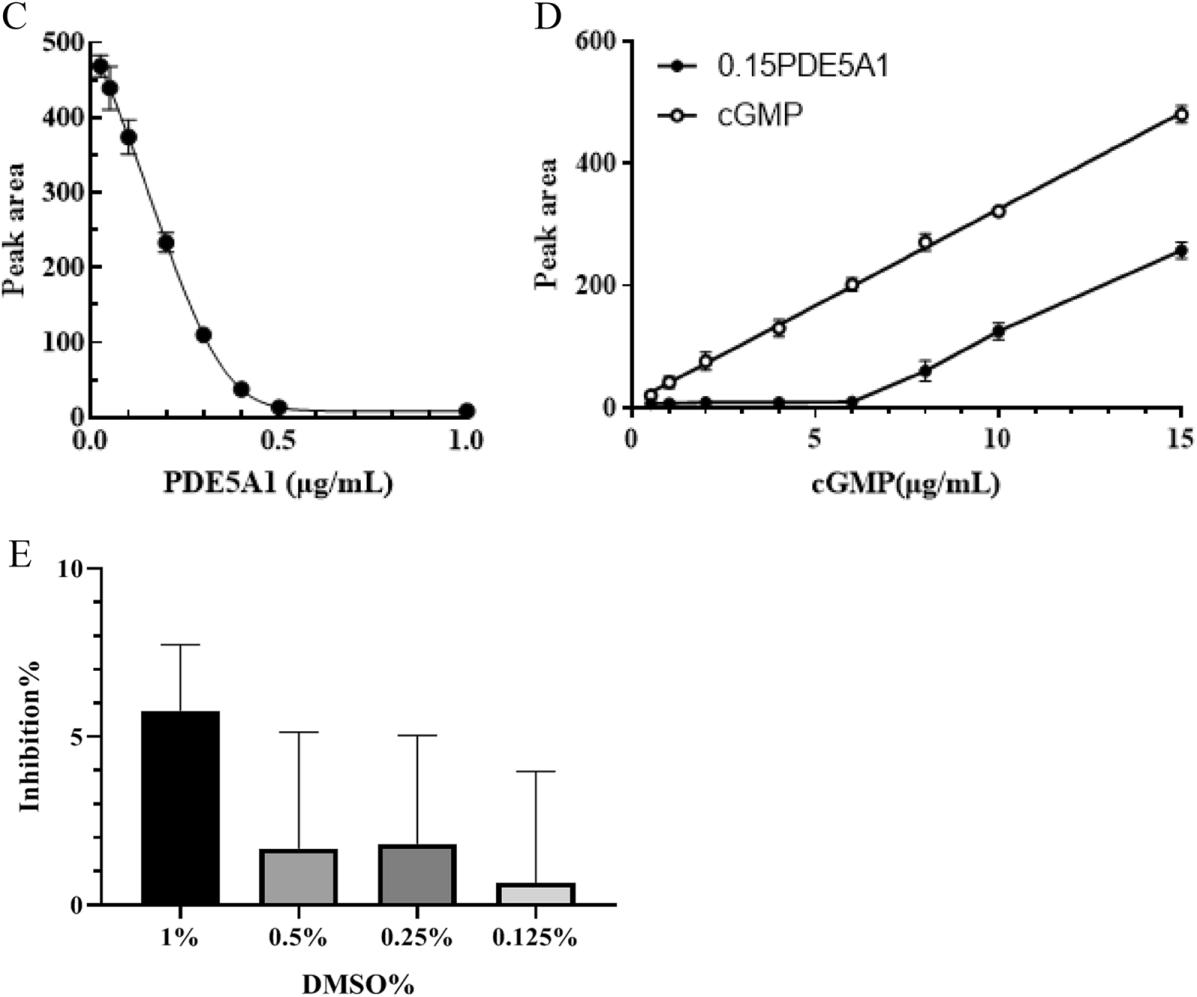
Schematic of HPLC based screening assay for PDE5A1-inhibitors. **A** Linear correlation of the standard curve for cGMP quantitatively detected by HPLC. The peaks from the bottom to the top showed the samples was prepared by 0.18 nM, 0.73 nM, 2.93 nM, 11.72 nM, 46.88 nM, 187.50 nM, 750.00 nM sildenafil. (B) The standard curve of cGMP. **C** Titration of PDE5A1 in the presence of 50 µg/mL of cGMP detected by HPLC. **D** Titration of cGMP in the presence of 0.15 µg/mL PDE5A1 (lower line) and the cGMP standard curve without PDE5A1 (upper line). **E** DMSO tolerance study for 0.15 µg/mL PDE5A1 in the presence of 7.5 µg/mL cGMP for 90 min reaction at 35 ℃

For developing the screening method for PDE5A1 inhibitors, the in vitro enzyme reaction and HPLC detection were combined. The decrease of cGMP peak area was detected by HPLC according to the hydrolysis of cGMP by PDE5A1 (Fig. 2). In the range of 0.21 ~ 150 μg/mL, cGMP standard curve fitted into well-defined linear relationship with the peak area (R^2^ > 0.999). In addition, it was confirmed that the detection of cGMP was not affected by the chemical composition within buffer (Fig. 3A, B), which limited the variables in this study. The concentrations of enzyme and substrate are important parameters in the inhibitor screening assay. The results in Fig. 3C showed that the PDE5A1 activity was linear with its concentration within range of 0.025 ~ 0.30 μg/mL. PDE5A1, with concentration of 0.15 μg/ml, could potentially satisfy the requirements of acceptable signal-to-noise ratio and error at the same time (Fig. 3C). As shown in Fig. 3D, with cGMP concentration lower than 6.0 µg/mL, linear relationship between PDE5A1 activity and its peak area was not observed due to unsaturated PDE5A1. As the PDE5A1 activity and its peak area showed high accordance with linear relationship with concentration higher than 6.0 μg/mL, 7.5 µg/mL was chosen and used as the optimized cGMP concentration for further experiments. Although the inhibition of PDE5A1 activity increased with the increase of DMSO concentration, the inhibition of PDE5A1 could be negligible when DMSO was below 0.5% (Fig. 3E). Taken together, the optimized assay conditions for PDE5A1 inhibitors screening were 0.15 µg/mL PDE5A1, 7.5 µg/mL cGMP, and 90 min reaction at 35 ℃.

### Evaluation of assay quality

**Fig. 4:**
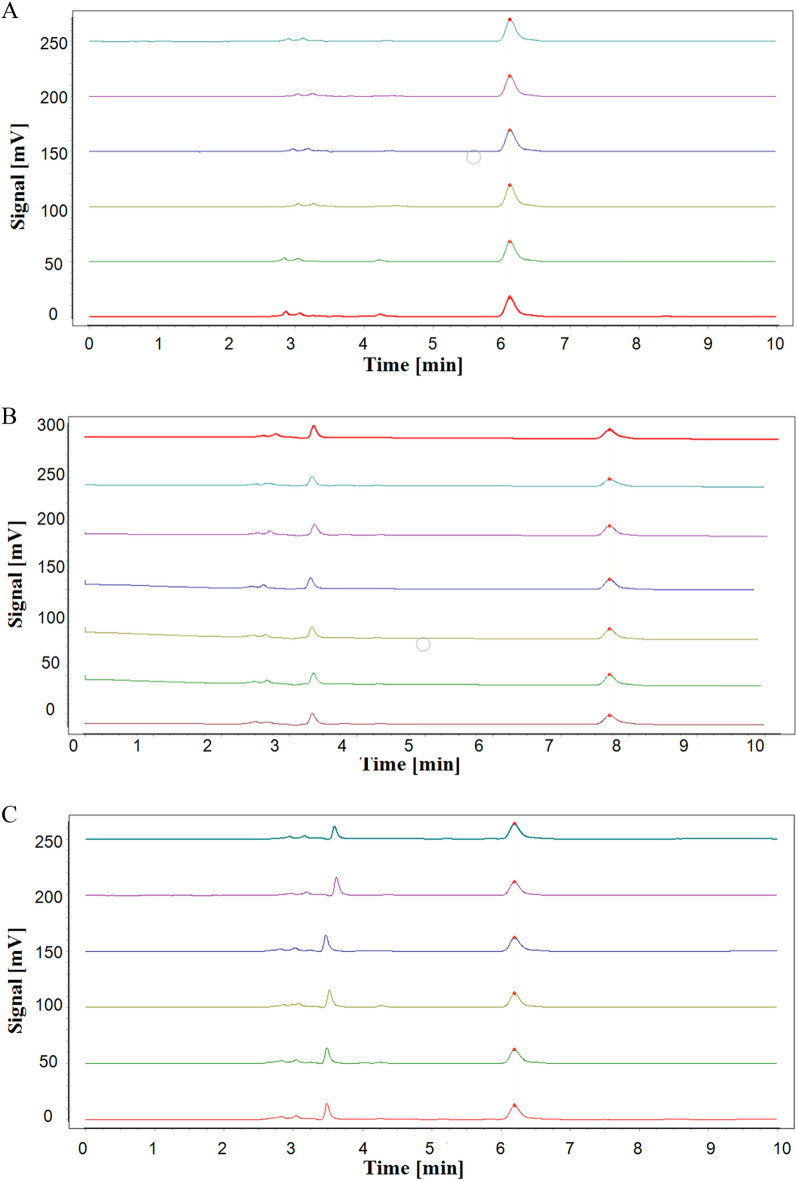

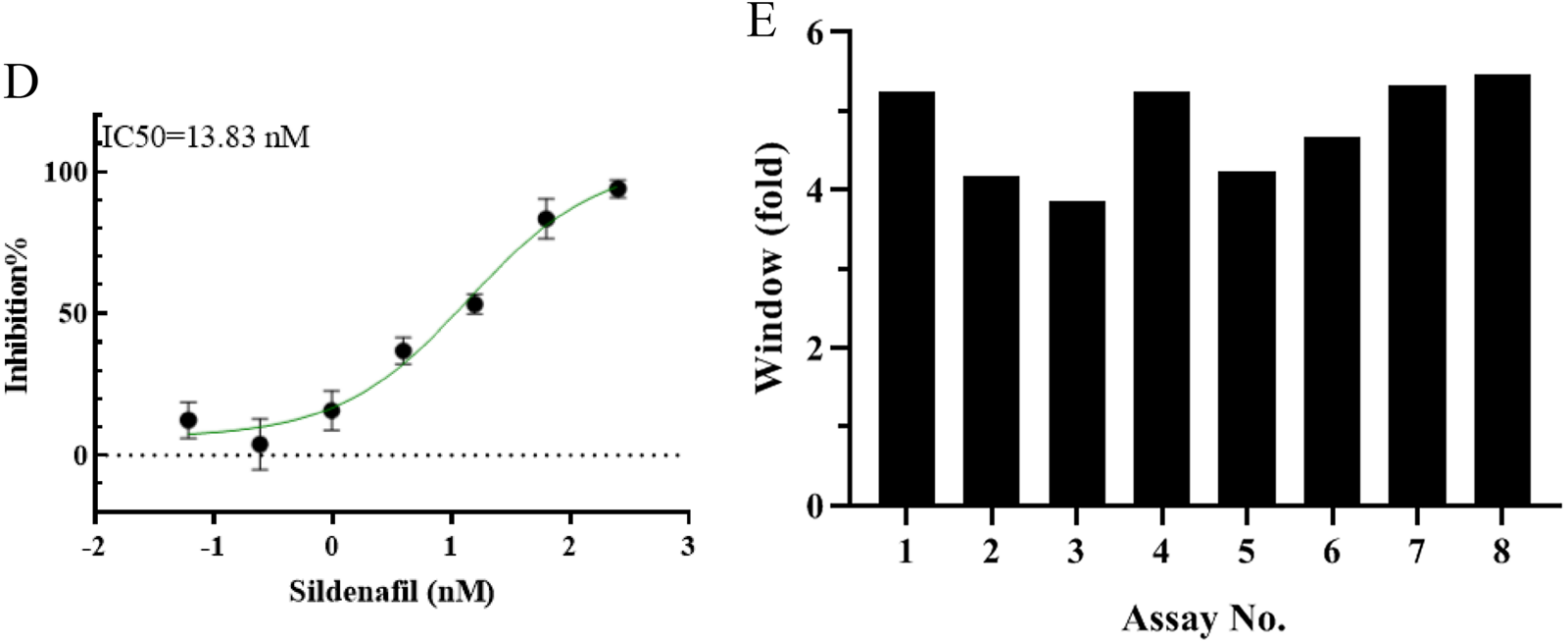
Quality evaluation of HPLC based PDE5A1-inhibitor screening assay. **A** The precision of HPLC-detected cGMP, which was 0.75 µg/mL, equal to the substrate concentration in the assay (n = 6). The pictures from the bottom to the top showed the the same sample was injected one to six. **B** The HPLC-detected analytes stability under 10 nM sildenafil in the assay within 24 h (n = 6). The pictures from the bottom to the top showed the peaks at 0 h, 2 h, 4 h, 8 h, 12 h, 24 h. **C** The reproducibility of the assay inhibited by 10 nM sildenafil (n = 6). The peaks showed 6 samples prepared in the same way. **D** The signal window of 8 independent experiments (n = 8). **E** Dose–response curves of sildenafil acting on PDE5A1- hydrolyzed cGMP production in the PDE5A1-inhibitor screening assay. IC_50_ = 14.22 nM ± 1.3 nM (n = 3)

The quality control (QC) was evaluated for this assay. The peak area of 7.5 μg/mL cGMP was repeated 6 times. The RSD of 1.6% indicated that the instrument had good precision (Fig. 4A). 10 nM sildenafil was used to inhibit the activity of PDE5A1, then the stability of the analytes was detected by HPLC within 24 h. The stability RSD obtained was 0.46%, indicating that the analytes kept stable for 24 h at room temperature, and no obvious impurity appeared to interfere the detection of cGMP (Fig. 4B). Further, the RSD was 1.6% after six repeated PDE5A1 assay inhibited by 10 nM sildenafil, which suggested that the assay was reproducible (Fig. 4C). The signal window (SW), as the ratio of positive control signal to negative control signal, was an important parameter for evaluating the stability of screening assay. The SW mean value collected from 8 independent experiments was 4.78, which was well within the range of requirement of S/B > 3 (Fig. 4D). Lastly, sildenafil IC_50_ to PDE5A1 was performed using this screening assay, and the result (IC_50_ = 14.22 nM ± 1.3 nM) was similar to reported IC_50_ data (5.6 nM) in other studies [28] (Fig. 4E). The QC results showed that the screening assay was stable, reproducible, reliable, and low cost for PDE5A1 inhibitors screening.

### PDE5A1 activity screening of 1–6

**Fig. 5:**
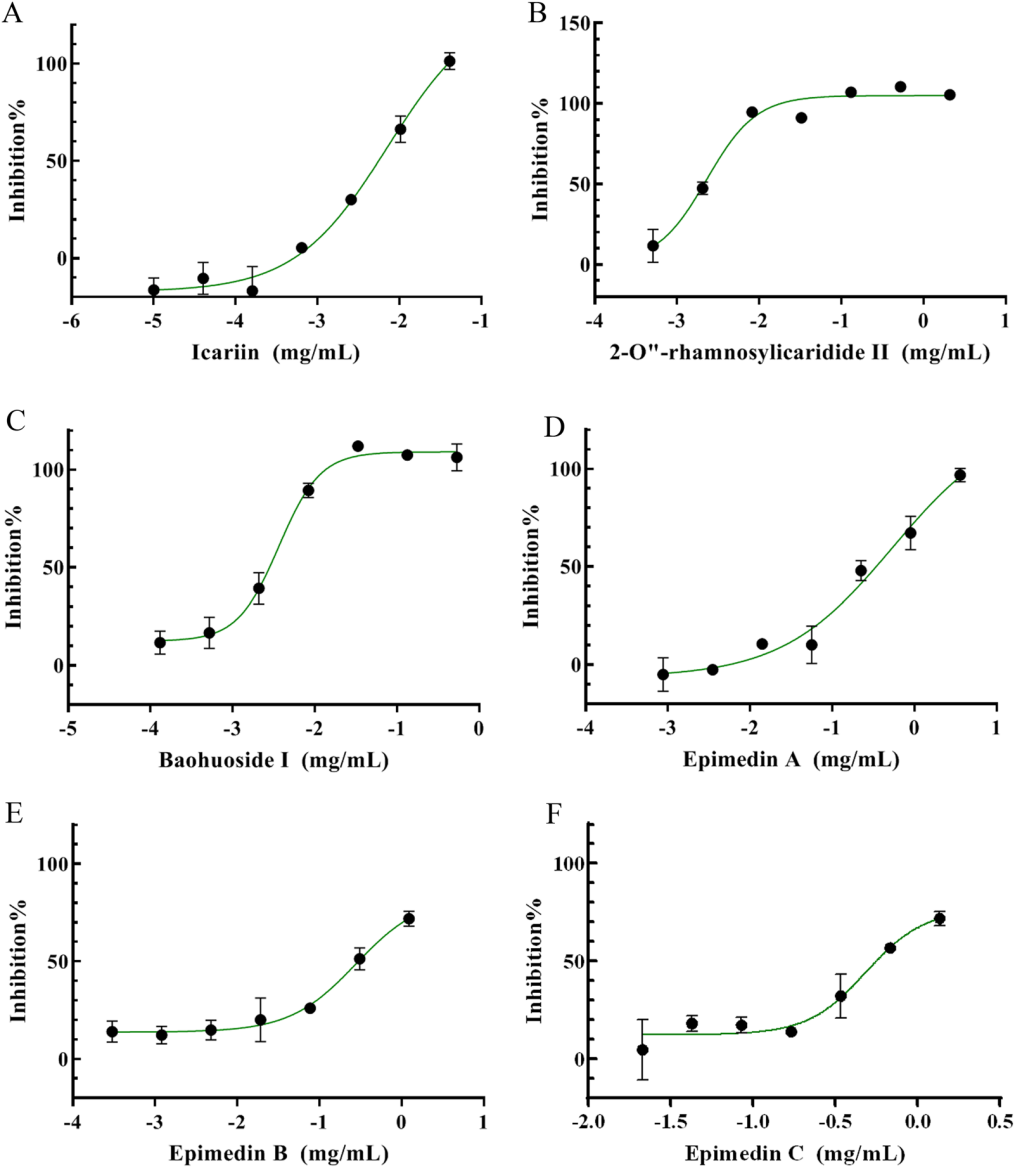
Dose–response curves of 8-isopentenyl flavonoids compounds in the PDE5A1-inhibitor screening assay. **A** Icariin. **B** 2-Oʹʹ-rhamnosylicaridide II. **C** Baohuoside I. **D** Epimedin A. **E** Epimedin B. **F** Epimedin C

**Fig. 6:**
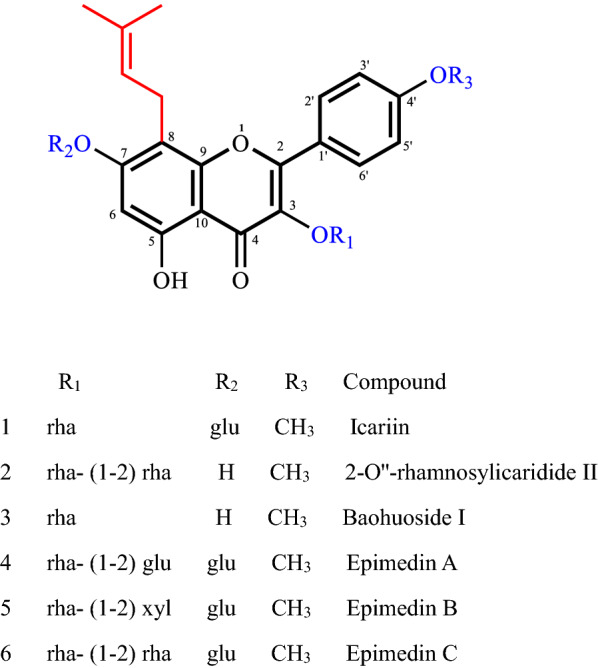
Structure of 8-isopentenyl flavonoids compounds from *Epimedium*

The dose-dependent inhibition behaviors of these compounds were analyzed by PDE5A1 inhibition assay. The IC_50_ of compounds 1, 2, 3, 4, 5, 6 were 8.28 μM, 3.23 μM, 5.48 μM, 408.6 μM, 357.6 μM and 682.0 μM, respectively (Fig. 5, Table 4). Among which, compounds 1, 2 and 3 showed sufficient inhibitory efficacy on PDE5A1 with compound 2 most potent (IC_50_ of 3.23 μM). Our research results showed that icariin inhibited PDE5A1 with IC_50_ of 8.28 μM, which was in line with other studies [11]. The high degree of consistency indicated that the novel HPLC-based detection method for PDE5A1 inhibitor was reliable and reproducible Fig. 6.

**Table 4:** 8-isopentenyl flavonoids compounds potency on PDE5A1

Compd	PDE5A1 (IC_50_ ± SD) (μM)
1	8.27 ± 0.74
2	3.23 ± 0.23
3	5.47 ± 0.56
4	408.60 ± 31.4
5	357.60 ± 34.2
6	681.90 ± 38.4
Sildenafil	0.0142 ± 0.0013

### CoMFA and CoMSIA models

#### Performance of CoMFA and CoMSIA models

The predictive capability (*q*^2^) of the model was found by the leave-one-out (LOO) method. The conventional correlation coefficient (*r*^2^), standard error of estimation (SEE) and *F* value were used to assess the non-cross-validated models. The four-component CoMFA model presented the most excellent values of parameters. This CoMFA model yielded a cross validated *q*^2^ of 0.578. The non-cross-validated PLS analysis gave the *r*^2^ of 0.998, *F* value of 1188.764 and the SEE of 0.066. The contributions of steric and electrostatic fields were 49.3% and 50.7% in this model, respectively. For CoMSIA model, we constructed various combinations of three fields in order to gain the best model, which was shown in Table 5. The combination of steric, hydrophobic and hydrogen bond accept fields presented the optimal result. This CoMSIA model, with the optimal number of components of 7, was characterized by the following statistics: *r*^2^ = 0.999, *q*^2^ = 0.505, SEE = 0.042 and *F* value = 1663.955. The contributions of steric, hydrophobic and hydrogen bond accept fields were 21.6%, 39.3% and 39.1% in this model, respectively. The statistical parameters for the CoMFA and CoMSIA models were given in Table 6.

**Table 5:** Summary of CoMSIA models for different combinations of three fields

Models	Fields^a^	*q*^2^	*r*^2^	SEE	*F* value	Components
1	SHA	0.505	0.999	0.042	1663.955	7
2	HAD	0.477	0.970	0.252	107.115	3
3	EHA	0.439	0.968	0.258	101.590	3
4	SAD	0.413	0.974	0.245	84.985	4
5	ESA	0.388	0.979	0.222	104.013	4
6	ESD	0.377	0.669	0.761	24.212	1
7	SHD	0.375	0.967	0.264	97.025	3
8	EAD	0.375	0.629	0.805	20.366	1
9	EHD	0.362	0.961	0.286	82.351	3
10	ESH	0.342	0.972	0.242	116.168	3

^a^*A* hydrogenbond acceptor, *D* hydrogenbond donor, *E* electrostatic, *S* steric, *H* hydrophobic

**Table 6:** Statistical indexes of CoMFA and CoMSIA models

	Cross-validated		Conventional		
	*q*^2^	Optimal comp	*r*^2^	SEE	*F* value
CoMFA	0.578	4	0.998	0.066	1188.764
CoMSIA	0.505	7	0.999	0.042	1663.960
Field distribution (%)					
	CoMFA		CoMSIA		
Steric	49.3		21.6		
Electrostatic	50.7				
Hydrophobic			39.3		
H-bondacceptor			39.1		

**Fig. 7:**
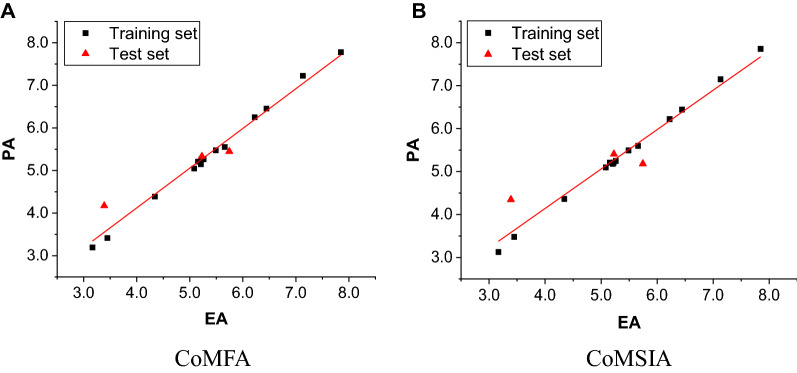
Predicted activities (PA) by CoMFA (**A**) and CoMSIA (**B**) models versus experimental activities (EA). Black filled square, compounds of the training set; red filled traingle, compounds of the test set

To validate the predictive ability of our 3D-QSAR model, biological activity of the three compounds of test set (compounds 4, 10 and 12 in Table 1) was predicted by both models. The actual activity and predicted activity data of the training set and test set were given in Table 7 for the best CoMFA and CoMSIA models. The testing results for the test set indicated that the CoMFA and CoMSIA models can be further used in new PDE5A1 inhibitors design. Simultaneously, Fig. 7 showed the correlation between the predicted pIC_50_ values and the experimental ones of all the compounds for the CoMFA and CoMSIA models. The CoMFA and CoMSIA models showed comparatively satisfactory predictive capability. It was worth noting that for two models, the predicted activity values of compound 4 showed a relatively big deviation (-0.958 and -0.784), compared with its experimental value. We speculate that the lack of the data with low activity value led to the result that the QSAR model failed to identify some key features of low activity compounds.

**Table 7:** Experimental activities (EA, − log IC_50_) and predicted activities (PA) and residual values for analyzed compounds according to CoMFA and CoMSIA

Compd	EA	CoMFA		CoMSIA	
		PA	Residual	PA	Residual
1	5.082	5.048	0.034	5.096	− 0.014
2	5.490	5.475	0.015	5.495	− 0.005
3	5.262	5.264	− 0.002	5.249	0.013
4^t^	3.389	4.173	− 0.784	4.347	− 0.958
5	3.447	3.415	0.032	3.482	− 0.035
6	3.166	3.195	− 0.029	3.129	0.037
7	5.658	5.553	0.105	5.600	0.058
8	6.444	6.456	− 0.012	6.444	0.000
9	7.131	7.227	− 0.096	7.149	− 0.018
10^t^	5.745	5.449	0.296	5.183	0.562
11	5.222	5.265	− 0.043	5.211	0.011
12^t^	5.229	5.335	− 0.106	5.408	− 0.179
13	5.155	5.208	− 0.053	5.208	− 0.053
14	5.208	5.148	0.06	5.183	0.025
15	4.342	4.389	− 0.047	4.364	− 0.022
16	7.847	7.779	0.068	7.857	− 0.011
17	6.222	6.254	− 0.032	6.221	0.001

*t* test set compounds

#### CoMFA and CoMSIA contour maps

Contour maps, as an efficient way to explore how to increase the biological activity of the compounds studied, can provide instructive suggestions for the structural optimization of compounds. These contour maps provided us some general insight into the nature of the receptor-ligand binding region [29].

**Fig. 8:**
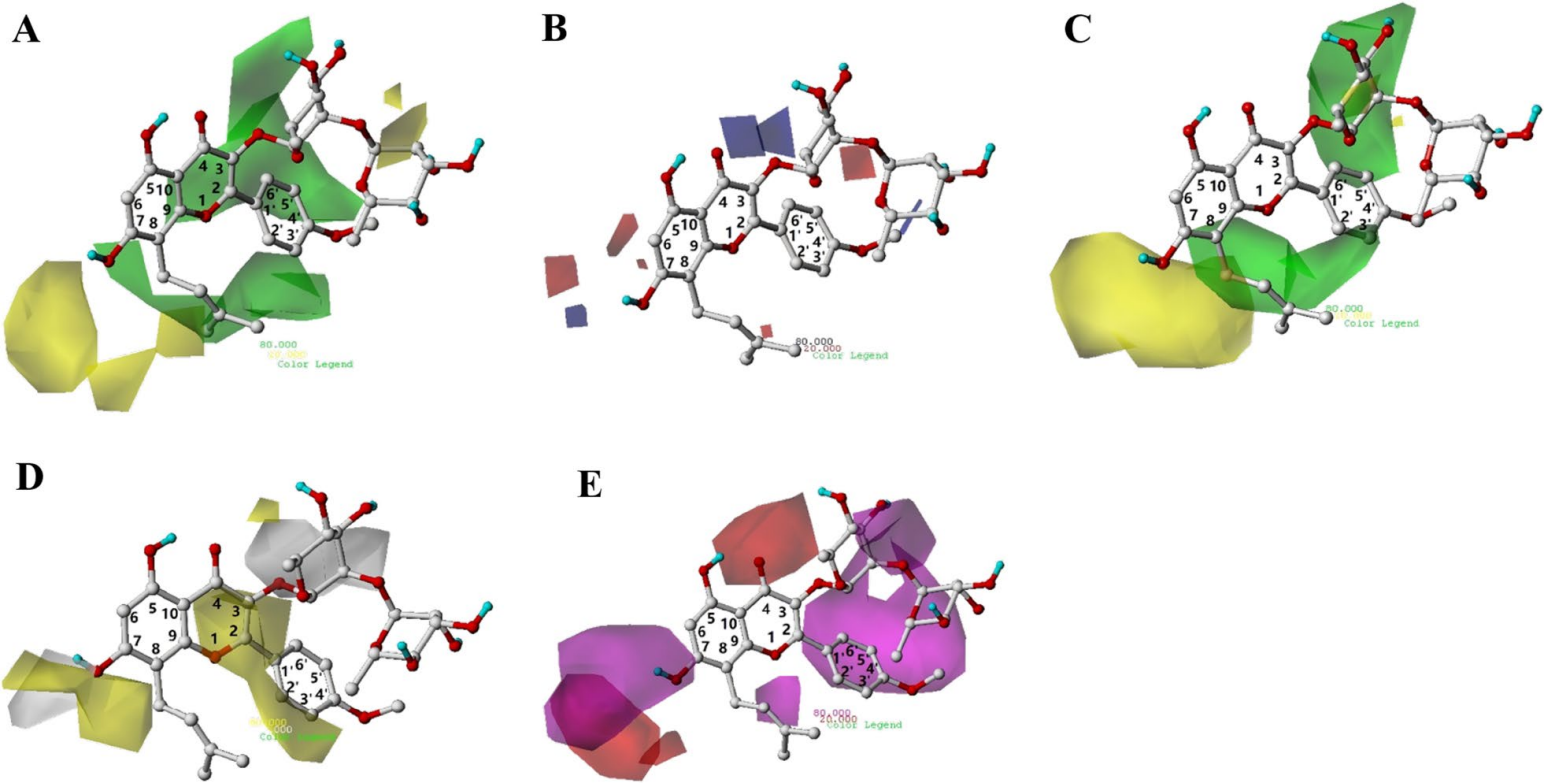
CoMFA contour maps of **A** favorable (green) and unfavorable (yellow) steric field, **B** electropositive (blue) and electronegative (red) fields. CoMSIA contour maps of **C** favorable (green) and unfavorable (yellow) steric field, **D** favorable (yellow) and unfavorable (white) hydrophobic field, **E** favorable (magenta) and unfavorable (red) hydrogen bond acceptor field

Contour maps for CoMFA and CoMSIA models were shown in Fig. 8. The most active compound **2** in the six compounds we studied, as the reference structure, was displayed in the map in aid of visualization.

##### CoMFA

The steric field contour map of CoMFA was shown in Fig. 8A. In the steric contour map of CoMFA, the larger steric features for groups near the green region or the less the ones near the yellow region were, the higher the bioactivity was. We can see that there is a large yellow polyhedron-like region around C7. This suggested that the replacement of substituent adjacent to C7 of ring A with bulky steric groups would be unfavorable for activity. This might explain why most of the compounds with a relatively minor substituent at this site, except compounds 5, 6 and 15 with a relatively bulkier group (e.g., glucose), exhibited improved potency. On the other hand, two large green polyhedrons in Fig. 8A indicate bulky substituents (e.g., isopentenyl group bound to C8 of ring A) are favorable for activity in these regions. The two substituents of ring B of compound 16 were located near the two green contours, which could partly explain the high activity of compound 16.

The electrostatic field contour map of CoMFA was shown in Fig. 8B. Blue region corresponds to areas where electropositive groups were favorable, while red area indicated that electronegative groups in the position increased the activity. According to the contour maps, there are two small blue polyhedrons around the C3 of ring C, which suggested the substituent with positive charge here would increase activity. A favorable negative red contour region was found in surroundings of oxygen atom of C3 substituent of ring C in compound 2. As the experimental data exhibited, the activity of compounds 2 and 3 was higher than compound 1 because the electronegative atom in the former substituent was closer to the red polyhedron.

##### CoMSIA

The CoMSIA steric contour map (Fig. 8C) was nearly in accordance with the field distributions of CoMFA map. A minor difference was that the yellow contour near ring B in CoMSIA steric contour map was absent.

Figure 8D displayed the contour map of hydrophobic field. For the hydrophobic field, yellow and white contours highlighted areas where hydrophobic and hydrophilic properties were favored. Two white contours were located near the C7 position of ring A and C3 position of ring C, indicating that incorporation of hydrophilic groups in these positions were favorable. For instance, Compounds 7, 8 and 9 showed improved inhibitory activity because of the hydrophilic group –OH of the C3 substituent present in the site. However, we could also see a yellow region around the white contour near ring A. Therefore, the length of hydrophobic or hydrophilic substituent should be selected with caution.

The CoMSIA hydrogen bond acceptor contour map was shown in Fig. 8E. In hydrogen bond acceptor contour map, the magenta and red contours identified favorable and unfavorable positions for hydrogen bond acceptors. Two large magenta polyhedrons near the C7 of ring A and the C4' of ring B were shown on the H-bond acceptor contour map, suggesting that a hydrogen bond acceptor substituent in the position may increase the activity of the compound. This was corroborated by the fact that the most active inhibitor 16 positions a substructure of sulfonyl group in the site. Similar to compound 16, compound 9, with a hydrogen bond acceptor group (-OH) at each of the two regions, displayed almost identical potency.

### 1 ~ 3 increased cGMP and decreased cytosolic calcium of CCSMCs

**Fig. 9:**
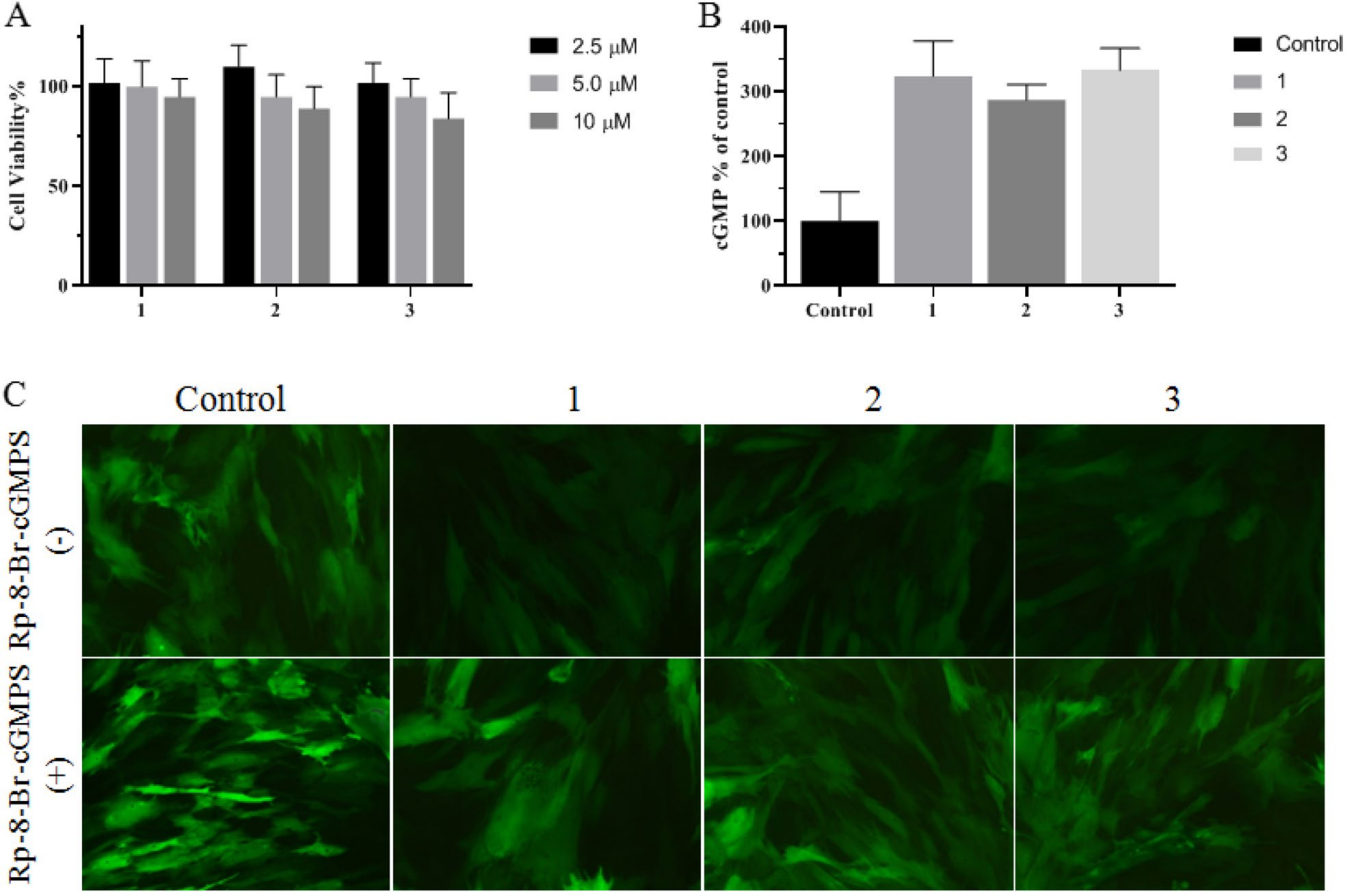
**A** CCSMCs cytotoxicity by compound 1 ~ 3 at 2.5 ~ 10 uM for 24 h. **B** The cGMP level of CCSMCs after treatment with compound 1 ~ 3 at 10 μM. **C** Fluorescence staining of calcium in CCSMCs by Fluo-4 AM after exposure to compound 1 ~ 3 at 10 uM for 30 min in the presence or absence of 10uM Rp-8-Br-cGMPS. Data were expressed as mean ± SEM. **p < 0:01 vs. Control group

cGMP plays a key role in regulating the relaxation of corpus cavernosum smooth muscle. In response to sexual stimulation, cGMP synthesis increases. 1 ~ 3 showed no significant cytotoxicity to CCSMCs below 10 μM (Fig. 9A), while increased the cGMP level (Fig. 9B). PKG exerts the strongest effect in cGMP-down-streamed-targeted kinase. cGMP, as a second messenger, activates PKG, which reduces cytoplasmic Ca^2+^ through the cascade reactions, thereby relaxing the corpus cavernosum artery and smooth muscle and causing hemoperfusion to induce penile erection. Blockade or inhibition of PDE5 activity causes an increase in cGMP levels in the intima and smooth muscle cells of the corpus cavernosum artery, leading to vasodilation and erection. 1 ~ 3 decreased the fluorescence intensity of cytoplasmic calcium in CCSMCs, and Rp-8-Br-cGMPS blocked the down-regulated calcium levels by 1 ~ 3, suggesting 1 ~ 3 regulated cytoplasmic calcium via PKG (Fig. 9C). The results were in line with other studies, the previous studies have shown that PDE5 inhibitors promote penile erection by activating PKG [30, 31]. It suggests 1 ~ 3 takes effect to CCSMCs by partly increasing cGMP, activating PKG and decreasing Ca^2+^, which may play an inhibitory role in the contraction of the corpus cavernosum smooth muscle.

## Conclusion

This study was the first to isolate and identify 6 main characteristic 8-isopentenyl flavonoids from PFES and to systematically detect their inhibitory activity on PDE5. The PDE5A1 inhibition assay was carried out by the enzyme reaction and HPLC detection, and this combination was not reported in previous literature. The HPLC detection was reproducible, reasonable, simple, and effective detection tool, and was supplementary to the previous expensive detection (See Additional file 1).

The alignment-based 3D-QSAR analyses were performed to study 8-isopentenyl flavonoids structural characteristic components as potent PDE5A1 inhibitors. Both CoMFA and CoMSIA models showed satisfactory results in terms of *q*^2^ and *r*^2^ values. The 10 CoMSIA models with different field combination were developed. Among them, the best CoMSIA model was generated from steric, hydrophobic and hydrogen-bond acceptor fields. Besides, contour maps of the two models provided enough information about structural requirement for biological activity. Taking compound **2** as the reference structure, the replacement of C8 of the ring A with bulky steric groups, C3 of the ring C with positive charge groups and C4' of ring B with a hydrogen bond acceptor substituent could increase activities. In contrast, the substitution of C7 of ring A with bulky steric groups or hydrophilic groups tended to decrease biological activity. The proposed models could be useful for us to understand the effects of different functional groups on inhibition activities, and in future, could provide us further guidelines for designing novel PDE5A1 inhibitors with higher potency. Furthermore, it was revealed that 1–3 inhibited the contraction of the corpus cavernosum smooth muscle by inhibiting PDE5 activity, increasing cGMP level, activating PKG and decreasing Ca^2+^. This study developed a new detection method for PDE5 inhibitors.

These results establish a novel HPLC assay for PDE5 inhibitors screening, provide a solid design for potent 8-isopentenyl flavonoids against PDE5, and reveal the potential mechanism by which the PFES rich in those characteristic PDE5 inhibitors is good at strengthening sexual function.

## Supplementary Information

**Additional file 1: Figure S1.** The figures of 1H NMR of Icariin. **Figure S2.** The figures of 1H NMR of 2-O''-rhamnosylicaridide II. **Figure S3.** The figures of 1H NMR of Baohuoside I. **Figure S4.** The figures of 1H NMR of Epimedin A. **Figure S5.** The figures of 1H NMR of Epimedin B. **Figure S6.** The figures of 1H NMR of Epimedin C.

## Data Availability

The datasets used and/or analysed during the current study are available from the corresponding author on reasonable request.

## Abbreviations

3D-QSAR: Three-dimensional quantitative structure–activity relationship
CCSMCs: Corpus cavernosum smooth muscle cells
cGMP: Cyclic guanosine monophosphate
CoMFA: Comparative molecular field analysis
CoMSIA: Comparative molecular similarity index analysis
^13^C NMR: Carbon nuclear magnetic resonance
ED: Erectile dysfunction
EtOAc: Ethyl acetate
^1^H NMR: Hydrogen nuclear magnetic resonance
HPLC: High-pressure liquid chromatography
IC_50_: Half maximal inhibitory concentration
LOO: Leave-One-Out
MS: Mass spectrum
NO: Nitric oxide
ONC: The optimum number of components
PDE5: Phosphodiesterase 5
PFES: The processed folium of *Epimedium sagittatum* Maxim
PKG: Protein kinase G
PLS: Partial least squares
RSD: Relative standard deviation
SAR: Structure–activity relationship

## References

[1] Mostafa T, Taymour M (2020). Gene polymorphisms affecting erectile dysfunction. Sex Med Rev.

[2] Chen Y, Zhang J (2019). PDE5 inhibitors as a medicinal guide drug in the treatment of erectile dysfunction. Nat J Androl.

[3] He J, Li X, Dai H (2019). The safety and efficacy of PDE5-inhibitors-vardenafil on treating diabetes mellitus erectile dysfunction: a protocol for systematic review and meta analysis. Medicine.

[4] Cai Z, Zhang J, Li H (2019). Two birds with one stone: Regular use of PDE5 inhibitors for treating male patients with erectile dysfunction and cardiovascular diseases. Cardiovasc Drugs Ther.

[5] Dhaliwal A, Gupta M (2022). PDE5 Inhibitors.

[6] Niu R (1989). Action of the drug Herba Epimedii on testosterone of the mouse plasma and its accessory sexual organ before and after processing. China J Chin Mater Med.

[7] Wang S, Qi M, Li F (2005). Effects of the crude and processed *Epimedium sagittatum*. maxim on castrated mice. Chin Tradit Patent Med.

[8] Gu S, Zhou R, Wang X (2018). Comparison of enhanced male mice sexual function among three medicinal materials. Andrologia.

[9] Chiu J, Chen K, Chien T (2006). *Epimedium brevicornum*. Maxim extract relaxes rabbit corpus cavernosum through multitargets on nitric oxide/cyclic guanosine monophosphate signaling pathway. Int J Impot Res.

[10] Chen X, Tang Z, Li X, Xie C, Lu J, Wang Y (2015). Chemical constituents, quality control, and bioactivity of *Epimedii Folium* (Yinyanghuo). Am J Chin Med.

[11] Dell'Agli M, Galli GV, Dal Cero E, Belluti F, Matera R, Zironi E, Pagliuca G, Bosisio E (2008). Potent inhibition of human phosphodiesterase-5 by icariin derivatives. J Nat Prod.

[12] Olotu FA, Agoni C, Soremekun O, Soliman MES (2020). The recent application of 3D-QSAR and docking studies to novel HIV-protease inhibitor drug discovery. Expert Opin Drug Discov.

[13] Nam KY, Choi NS, Han CK, Ahn SK (2012). Identification of chalcones as potent and selective PDE5A1 inhibitors. Bioorg Med Chem Lett.

[14] Sybyl-X Molecular modeling software packages, Version 20 TRIPOS associates Inc St Louis MO USA: 2012.

[15] Bush BL, Nachbar RB (1993). Sample-distance partial least squares: PLS optimized for many variables, with application to CoMFA. J Comput Aided Mol Des.

[16] Wold S (1978). Cross-validatory estimation of the number of components in factor and principal components models. Technometrics.

[17] Liu R, Li A, Sun A, Cui J, Kong L (2005). Preparative isolation and purification of three flavonoids from the Chinese medicinal plant *Epimedium koreamum*. Nakai by high-speed counter-current chromatography. J Chromatogr A.

[18] Kang S, Kang Y, Lee M (1991). Flavonoids from *Epimedium koreanum*. J Nat Prod.

[19] Ma A, Qi S, Xu D, Zhang X, Daloze P, Chen H (2004). Baohuoside-1, a novel immunosuppressive molecule, inhibits lymphocyte activation in vitro and in vivo. Transplantation.

[20] Kim E, Kim M, Kang H (2008). Flavonol glycosides with antioxidant activity from the aerial parts of *Epimedium koreanum* Nakai. Nat Prod Sci.

[21] Fiorito J, Vendome J, Saeed F (2017). Identification of a novel 1, 2, 3, 4-tetrahydrobenzo [b][1,6] naphthyridine analogue as a potent phosphodiesterase 5 inhibitor with improved aqueous solubility for the treatment of alzheimer's disease. J Med Chem.

[22] Zhang L, Seo JH, Li H (2018). The phosphodiesterase 5 inhibitor, KJH-1002, reverses a mouse model of amnesia by activating a cGMP/cAMP response element binding protein pathway and decreasing oxidative damage. Br J Pharmacol.

[23] Abdel-Halim M, Sigler S, Racheed NAS (2021). From celecoxib to a novel class of phosphodiesterase 5 inhibitors: trisubstituted pyrazolines as novel phosphodiesterase 5 inhibitors with extremely high potency and phosphodiesterase isozyme selectivity. J Med Chem.

[24] Casarini L, Riccetti L, Limoncella S (2019). Probing the effect of sildenafil on progesterone and testosterone production by an intracellular FRET/BRET combined approach. Biochemistry.

[25] Ahmed NS, Gary BD, Tinsley HN (2011). Design, synthesis and structure-activity relationship of functionalized tetrahydro-β-carboline derivatives as novel PDE5 inhibitors. Arch Pharm.

[26] Lin C (2004). Tissue expression, distribution, and regulation of PDE5. J Impot Res.

[27] Lin C, Lin G, Xin Z (2006). Expression, distribution and regulation of phosphodiesterase 5. Curr Pharm Des.

[28] Lin C, Chow S, Lau A (2002). Human PDE5A gene encodes three PDE5 isoforms from two alternate promoters. Int J Impot Res.

[29] Babu S, Sohn H, Madhavan T (2015). Computational analysis of CRTh2 receptor antagonist: a ligand-based CoMFA and CoMSIA approach. Comput Biol Chem.

[30] Muzaffar S, Shukla N, Srivastava A (2005). Sildenafil citrate and sildenafil nitrate (NCX 911) are potent inhibitors of superoxide formation and gp91phox expression in porcine pulmonary artery endothelial cells. Br J Pharmacol.

[31] Shukla N, Rossoni G, Hotston M (2009). Effect of hydrogen sulphide-donating sildenafil (ACS6) on erectile function and oxidative stress in rabbit isolated corpus cavernosum and in hypertensive rats. BJU Int.

