# Supplementary material for: Isolation, bioassay and 3D-QSAR analysis of 8-isopentenyl flavonoids from Epimedium sagittatum maxim. as PDE5A inhibitors
Source: Chin Med. 2022 Dec 31;17:147. doi: 10.1186/s13020-022-00705-5 (PMC9805685; doi:10.1186/s13020-022-00705-5)
Supplement: Supplementary file 1 — Additional file 1: Figure S1. The figures of 1H NMR of Icariin. Figure S2. The figures of 1H NMR of 2-O''-rhamnosylicaridide II. Figure S3. The figures of 1H NMR of Baohuoside I. Figure S4. The figures of 1H NMR of Epimedin A. Figure S5. The figures of 1H NMR of Epimedin B. Figure S6. The figures of 1H NMR of Epimedin C. [file 13020_2022_705_MOESM1_ESM.docx]

**Figure S1. The figures of ^1^H NMR of Icariin**

**Figure S2. The figures of ^1^H NMR of 2-O''-rhamnosylicaridide II**

**Figure S3. The figures of ^1^H NMR of Baohuoside I**

**Figure S4. The figures of ^1^H NMR of Epimedin A**

**Figure S5. The figures of ^1^H NMR of Epimedin B**

**Figure S6. The figures of ^1^H NMR of Epimedin C**
